# Negotiating social norms, the legacy of vertical health initiatives and contradicting health policies: a qualitative study of health professionals’ perceptions and attitudes of providing adolescent sexual and reproductive health care in Arusha and Kilimanjaro region, Tanzania

**DOI:** 10.1080/16549716.2020.1775992

**Published:** 2020-06-26

**Authors:** Sara Bylund, Mats Målqvist, Nosim Peter, Sibylle Herzig van Wees

**Affiliations:** a UGHRIS - Uppsala Global Health Research on Implementation and Sustainability, Department of Women’s and Children’s Health, Uppsala University, Uppsala, Sweden; b Health Department, Evangelic-Lutheran Church of Tanzania, Arusha, Tanzania

**Keywords:** Adolescent sexual and reproductive health, Tanzania, health providers’ attitudes, qualitative research, providing health care

## Abstract

**Background:**

Adolescents in Tanzania are at risk of many health problems attributed to limited access to quality sexual and reproductive health services. Health professionals are a crucial part of service delivery, and their perspective on providing care is important in understanding the barriers that hamper access to sexual and reproductive health services for adolescents. Better understanding these barriers will support the development of more effective interventions. This paper explores these perspectives in view of the health-policy context that surrounds them.

**Objective:**

This study has aimed to explore and understand health professionals’ perceptions and attitudes regarding the provision of adolescent sexual and reproductive health care in a selected national sexual and reproductive health programme in the Arusha region and Kilimanjaro region, Tanzania.

**Methods:**

A qualitative cross-sectional interview design was applied. Sixteen in-depth interviews were conducted with health professionals and community health workers. Data was analysed following inductive thematic analysis.

**Results:**

Four main themes are identified in the data: concern about the stigma directed towards adolescents; over-medicalisation of services; difficulty involving adolescent males; and ambiguous policies and contradictory messages. The findings suggest that health professionals providing care in the current adolescent sexual and reproductive health programme must navigate the legacy of vertical health programmes as well as contradicting views and messages that are influenced by social norms, by uncertainties about current laws and by statements from political leaders.

**Conclusions:**

The findings suggest that future research, policies and health programmes should consider the perspectives of health professionals and their challenges in delivering care for adolescents to help improve the understanding of how to effectively and sensitively implement sexual and reproductive health programmes for adolescents.

## Background

Sexual and reproductive health is acknowledged as a human right and is a key component of the physical and emotional health of all individuals [1]. Progress has been made to improve sexual and reproductive health outcomes since the 1994 International Conference on Population and Development, but adolescents, defined by the World Health Organization (WHO) as people between 10 and 19 years old [2], continue to be disproportionately affected in terms of their access to sexual and reproductive health services [2]. In low- and middle-income countries, the leading causes of poor sexual and reproductive health outcomes are related to child marriage, teenage pregnancy, sexually transmitted infections (STIs) and human immunodeficiency virus (HIV) transmission [3–5].

Tanzania has one of the youngest populations in Africa: 44% of the population is under the age of 15 [6], and adolescents between 15–24 years account for 20% of the total population [7]. Despite reductions in the global total fertility rate and Tanzania’s national fertility rate [8], Tanzania’s adolescent fertility rate (age 15–19) increased from 116 to 132 births per 1000 women from 2010–2016, one of the highest adolescent fertility rates in the world [9]. While adolescence in Tanzania has been associated with high levels of adolescent fertility, a high prevalence of child marriage and low levels of knowledge about STIs, adolescent access to quality sexual and reproductive health care remains very low [10]. Among unmarried girls, 64% aged 15–19 years, who are sexually active, have unmet contraception needs [11]. This limited access to sexual and reproductive health services increases the risks of unplanned pregnancy, unsafe abortion and STIs [12,13]. Adolescent pregnancy for example, can be both a cause and a consequence of school dropouts [14], hindering entry into the labour market and reducing adolescents’ opportunities for emotional and physical growth, including life skills and self-confidence [14,15]. Moreover, complications related to adolescent pregnancy are the leading cause of death among adolescent girls in the world [16].

Adolescents experience barriers to accessing sexual and reproductive health services at the economic, political, and socio-cultural levels [1]. There are political and legal restrictions to accessing contraceptives in a number of countries. According to the World Population Policies Database [17], 35 countries have one or more policies that restrict access to contraceptive services. Many countries have criminal laws that prohibit or restrict access to abortion services, leading to high rates of unwanted pregnancies and maternal mortality from unsafe abortions [1,2]. National criminal laws that provide restrictions and censor sexuality-related information often define such information as ‘obscene’, ‘indecent’, or ‘against public morals’, which limits the effective distribution of health information. As these laws are unclear and do not specifically define what they cover, their interpretation is challenging for people living in these communities [18]. This challenge may, in turn, affect healthcare professionals who are part of those communities. For example, if women and health care providers do not know what the law prohibits, certain legal services may end up being denied [19].

In Tanzania, the Adolescent Health and Development Strategy 2018–2022 [10] and the National Health Policy 2017 [20] endorses adolescents’ access to sexual and reproductive health services, yet there is some confusion about how well policies and other legislative frameworks are being implemented [10]. Information on which policies currently govern adolescent-friendly health services, defined as services that need to be acceptable, accessible, comprehensive, effective, efficient, equitable and appropriate for young people [21], have lacked clarity according to service providers in Tanzania [22]. Furthermore, sexual and reproductive health policies for adolescents have been criticized for lacking coordination, as they span multiple Tanzanian ministries [22]. This has had consequences for adolescents in Tanzania, for example, the minimum standards for provision of adolescent-specific health services during delivery has been weak [20]. Moreover, definitions of adolescence have been lacking in the judiciary, rather they are submerged into broader categories like children and youth, which leaves the health needs for adolescents unrecognized [10].

Stigma towards adolescents seeking sexual and reproductive health care is a well-researched barrier to accessing adolescent sexual and reproductive health [1,23–25]. Adolescent sexuality is stigmatized in many communities, particularly when associated with STIs, including HIV [2,4]. In Tanzania, sexuality education is mostly discouraged by parents or guardians due to socio-cultural factors that restrict parents from communicating with their children about sexual and reproductive health [26]. Unmarried women accessing sexual and reproductive health services are particularly vulnerable to stigma [1]. Research from Tanzania suggests pervasive community-level stigma surrounding the sexual and reproductive health of unmarried adolescents, prompting shame, social and physical isolation, verbal harassment and physical punishment [27]. The stigmatization of adolescents by health care professionals and in communities has been found to significantly impact their access to sexual and reproductive health services in low- and middle-income countries. Adolescents do not seek or obtain care because of fear, experiences of shame, lack of confidentiality and unfriendly staff, leading to unwanted pregnancies and difficulties obtaining contraceptives at health facilities [23–25].

Healthcare professionals play a crucial role in supporting and guiding adolescents in their sexual and reproductive health decisions [28]. Community members and service providers in Tanzania have reported that it is inappropriate for girls aged 10–18 to access sexual and reproductive health services, especially contraceptives [29]. Health professionals in Tanzania have also been reported to have low knowledge about the sexual and reproductive health needs of adolescents [29], and health professionals’ negative attitudes have been cited as a further barrier to adolescents’ access to sexual and reproductive health services [27,29–31]. Findings from Tanzania further suggest that access to family planning services for adolescents is hampered by health providers’ age restrictions and personal biases, many of which are based on unfounded medical justifications not in compliance with the Tanzanian National Family Planning Programme guidelines and standards [12,29].

Consequently, the conceptual framework that guides this inquiry explores the relationship between the above-described uncertainties around the legal and policy framework that informs the healthcare providers’ mandate to provide sexual and reproductive health services to adolescents, and concerns expressed in the literature about the stigma experienced by adolescents seeking sexual and reproductive health services when seeking health care. This research has aimed to understand this dynamic by gaining a deeper understanding of the perceptions and attitudes of health professionals providing adolescent sexual and reproductive health services in the context of the Arusha, Hai, and Moshi district in Tanzania.

## Methods

### Study aim, design and setting

This qualitative study explores the perceptions and attitudes of health professionals providing adolescent sexual and reproductive health care within a national adolescent sexual and reproductive health programme (2018–2020) in the Arusha, Hai, and Moshi districts. This programme is implemented by the Evangelical-Lutheran Church in Tanzania (ELCT), supported by the Church of Sweden, which aims to improve access to sexual and reproductive health information and to provide education and services to adolescents at ten hospitals in ten districts in Tanzania. The ELCT is the second largest church in Tanzania, with 26 dioceses throughout the county. The church is responsible for several national programmes and interventions and provides several public health services such as health- and education services. By implementing a sexual and reproductive health programme for adolescents, the ELCT aims to complement the government’s National Road Map Strategic Plan to Improve Reproductive, Maternal, New-born, Child & Adolescent Health in Tanzania (2016–2020) [32]. Three rural district hospitals were randomly selected by SB and NP for this study: Marangu Lutheran Hospital in Arusha district (Arusha region), Machame Lutheran Hospital in Hai district (Kilimanjaro region) and Selian Lutheran in Moshi rural district (Kilimanjaro region). Two offices at the ELCT Health Department in the city of Arusha were selected for data collection with health professionals working with adolescent sexual and reproductive health at the community level.

### Sampling and participants

Purposive sampling was applied to ensure that data was collected with participants matching the specific inclusion and exclusion criteria [33]. Inclusion criteria were that participants should be health professionals, aged 18 years and above, responsible for any sexual and reproductive health service for adolescents or should work within the current programme under the ELCT. Furthermore, all participants had to have been working at the ELCT for at least 1 year. Individuals were excluded if below 18 years of age and if having less than 1-year work experience with sexual and reproductive health care and activities for adolescents. There was an element of snowball sampling involved, whereby some key persons were referred to by those involved in the health programme. A heterogeneous sample [33] was chosen to include individuals with different occupations, education, gender, and work areas to gain diversity in perceptions and experiences. Furthermore, health professionals working at the community level, as well as at the facility level, were included to gain further diversity. A description of the participants is provided in Table 1. Participants included key persons affiliated with the delivery of health services within the adolescent sexual and reproductive health programme, including 9 health professionals working at the district hospitals and 7 community health workers. Eleven females and 5 males are included in the sample, but to protect anonymity, the gender of each participant is not revealed. All participants were contacted by phone or email or were approached at their offices by NP, who is a social worker at the ELCT Health Department with knowledge of health professionals’ roles at the hospitals and ELCT offices. Employees at the hospitals supported NP with the selection of participants who fit the inclusion and exclusion criteria for the study. Participants working at the ELCT Health Department were selected by SB and NP based on inclusion and exclusion criteria.

**Table 1.** Summary of characteristics of participants.

Position	Discipline	Workplace/position
Nurse	Nursing	District hospital
Clinical officer	Medicine	District hospital
Social worker	Social work	District hospital
Nurse	Nursing	District hospital
Midwife	Nursing	District hospital
Midwife	Nursing	District hospital
Social worker	Community development	District hospital
Medical doctor	Medicine	District hospital
Medical doctor	Medicine	District hospital
Nurse/social worker	Nursing	Community health worker
HIV officer	Clinical medicine	Community health worker
HIV officer	Nutrition	Community health worker
Data clerk	Sociology	Community health worker
Psychologist	Psychology	Community health worker
Social worker	Sociology	Community health worker
Program officer	Medicine	Community health worker

### Data collection

Confidentiality, anonymity and the dissemination strategy were communicated in information sheets and verbally explained. Each participant was informed about their right to withdraw consent or participation at any stage of the interview or study. Data was collected by SB on audio tape using in-depth interviews to understand health professionals’ perceptions of delivering adolescent sexual and reproductive health services [34]. The topic guide (Annex 2) included some open-ended questions but primarily focused on allowing health professionals to describe examples of providing care to adolescents. Probing was used to explore topic themes covered in the literature – for example, their experience of policy and laws and issues related to stigma. One new topic was added after two interviews, which was the role of men when providing services to women.

The topic guide was pilot-tested by SB one time with staff at the ELCT Health Department office and was revised after feedback from NP. The pilot interview was not included in the analysis or results. Data collection was conducted in private rooms at the hospitals or at the participants’ respective offices. Interviews were conducted in English when participants felt comfortable and able to do so. A Swahili interpreter was present in three interviews because of language restrictions. The interviews lasted from 21 minutes to 1 hour and 21 minutes. Data saturation was reached when no additional data was being found in successive interviews [33]. In the 14th interview, no new themes were being generated, and it was decided that the data collection had reached saturation. Data collection then continued for two further interviews to ensure and confirm that no new topics emerged.

### Data analysis

Data was analysed by employing qualitative thematic analysis inspired by Saldaña’s manual for qualitative research [35]. The coding process was performed in NVivo 12. This study is situated in the constructivist paradigm [36,37] to understand the social world of the participants through interpretation of the attitudes and perceptions of health professionals in the context of their practice. An inductive approach was therefore used to analyse the data. The interviews were transcribed verbatim by SB. Transcripts were initially read through several times to gain an understanding of the material by paying attention to repeated patterns of meanings in the interviews. SB performed three rounds of coding. SH performed two rounds of coding. Codes were compared throughout the process, and a coding tree was agreed upon. The procedure of coding and categorizing was done iteratively before the final themes were created. An example of the coding tree is presented in Table 2.

**Table 2.** Example of moving from text via codes to category and theme.

Text	Codes	Category	Theme
If you mention sexual and reproductive health in the hospital, somebody will just refer you to a gynaecologist or somebody and … Not for a young person, unless you are pregnant or you have problem with, yeah … An STI. But not those general services for young people. These services [testing for STIs] are the most important because you found that, for instance, many adolescents need these services so that we can prevent those from having sexual transmitted diseases such as HIV … It is very important, as we are aiming to minimize the number if people with STIs and HIV …	Non pregnant adolescents do not have access to services STI testing for adolescents Testing for STIs most important service HIV positive adolescents can access health services	Prioritize young people with HIV in provision of health care	Over-medicalisation of services

### Trustworthiness

The methods section transparently describes each step of the research process to strengthen the dependability of the research process. SB took extensive notes and wrote a diary to ensure reflexivity was practiced during the data collection. Diary notes were discussed during the analysis procedures to reduce inter-subjectivity and to secure confirmability and credibility. Several rounds of coding by SB and SH were further conducted to increase credibility and confirmability.

The above description of this study’s methods enables the results to transfer more easily to similar settings. However, for the results to be transferable to a more diverse sample, a selection of various facilities would be required.

## Results

Thematic analysis resulted in four themes: concern about the stigma directed towards adolescents; over-medicalisation of services; difficulty involving adolescent males; and ambiguous policies and contradictory messages. Each theme describes the categories within that theme and uses a selection of quotations to illustrate the findings. A summary of the results is presented in Table 3.

**Table 3.** Summary of categories and themes.

Themes	Categories
Concern about the stigma directed towards adolescents	Prejudice against unmarried adolescents Senior staff having the most negative attitudes Worry about adolescents not seeking care
Over-medicalisation of services	Prioritization of pregnant adolescents in the provision of health care Prioritization of young people with HIV in the provision of health care Perception of testing for STIs as the most important service
Difficulty involving adolescent males	Concerns for men’s underuse of health services Pregnancy-focus that excludes men Suggestion that men link service to HIV service Lost opportunity if health care professionals fail to involve men
Ambiguous policies and contradictory messages	Uncertainty caused by president’s statement Fear of going against the law Strong legacy of discriminative policies Fear of challenging social and cultural norms

### Concern about the stigma directed towards adolescents

The need for friendlier attitudes among health care professionals and stigma towards adolescents accessing sexual and reproductive health care was clearly voiced by interviewees. Unmarried adolescents under 18 years experience many stigmas when they want to access sexual and reproductive health care:

The health worker starts yelling at them [the unmarried adolescent girls] ‘you’re supposed to be in school, why are you coming to ask for condom or contraceptives?’. [Interview 6, nurse]

The absence of friendly attitudes among health care professionals was perceived to be more common among senior health care professionals:

When the age [difference] is too wide, the health provider becomes like their parents instead of becoming [the adolescents’] friends. And … There are so many things that they can be judgmental for, which makes the youth not to open up. [Interview 16, programme officer]

Young health professionals were, therefore, commonly perceived as the most suitable counsellor, as they are more likely to act as peers, keep sexual issues private, and understand the challenges related to sexual and reproductive health that adolescents may face.

Interview data suggests that some health professionals are concerned about adolescents’ future health-seeking behavior when encountering judgmental health professionals:

They [health professionals] say that the kids are too young, they are not married: ‘Why do you come here seeking this … What are you doing here?’. If they ask such questions, they [the adolescents] will never come again. […] So then they tell others [peers], ‘Don’t go there!’ [Interview 3, midwife]

These responses show that stigma is perceived as a problem and that concern exists for these adolescents and their future health-seeking behavior.

### Over-medicalisation of services

The second theme that emerged was that sexual and reproductive health services primarily have a medical focus. Health professionals in the sexual and reproductive health programmes for adolescents primarily prioritize adolescents who test for HIV or who show STI symptoms:

For young people testing for HIV is the most important service. And for … Like syphilis or so, these sexually transmitted diseases, to test them. [Interview 4, nurse]

The second focus of the service is adolescent pregnant women, regardless of their marital status:

If you mention sexual and reproductive health in the hospital, somebody will just refer you to a gynaecologist or somebody and … [These services are] Not for a young person, unless you are pregnant, or you have problem with yeah … An STI. But not those general [sexual and reproductive health] services for young people. [Interview 12, HIV-officer]

At the hospitals, certain clubs are available whose clinics allow only adolescents and young people to attend. However, the clubs do not focus on comprehensive sexual and reproductive health; instead, the focus is on young people living with HIV only:

Somehow, in a way, it becomes very difficult for us to provide *effectively* sexual and reproductive health services, because sometimes here we are only … I mean saving those adolescents of HIV/AIDS. [Interview 1, female, social worker]

The above interview response further illustrates the concerns health professionals raised with this approach because it hinders them from delivering comprehensive services. Participants further expressed concern that a focus on STIs and pregnancy leaves adolescents with the impression that they are not welcome to receive sexual and reproductive health services unless they are pregnant or show symptoms of a disease.

### Difficulty involving adolescent males

The third theme that the data analysis generated was concern over challenges involving adolescent males in the healthcare services provided for adolescents. One of the main problems appears to be the perception of the service, which is linked to the preceding theme:

When they [the adolescents] hear about sexual and reproductive health, they think only about those people who are going for clinic services, for their kids. For being pregnant. Like that. But even for their fathers, they don’t go there. They think it’s only for pregnant women and those who are having small kids, but not for everybody. [Interview 14, psychologist]

The health professionals expressed concerns about adolescent males not using the sexual and reproductive health services available. The adolescent males who do attend were mainly there for HIV-testing with their pregnant partner; they remain excluded from other sexual and reproductive health services:

Most of them they come nowadays because they have to check for HIV during pregnancy, but in the second visit they … They disappear. [Interview 10, nurse]

Thus, adolescent males are perceived to be lacking knowledge about sexual and reproductive health in general, which further makes both them and their future children unaware of issues related to the topic. The absence of men in these services makes it difficult to discuss shared responsibilities in family planning as well as raising awareness of methods and rights:

I think it is not fair that the family planning in the family … That the women are only responsible for the family planning, but men not. No, this is very painful. [Interview 7, midwife]

Health professionals perceive the involvement of adolescent males to be important but struggle to involve them in a health setting that prioritises HIV interventions and pregnancy services.

### Ambiguous policies and contradictory messages

The fourth theme that the analysis generated relates to health professionals’ struggle to deal with ambiguous policies and contradictory messages. One of the main pillars of the national sexual and reproductive health programme is to prioritize and improve access to family planning for adolescents. Despite this health programme, health professionals interviewed in this study repeatedly talked about the challenges they experience due to mixed messages concerning their ability to provide care to adolescents from the health sector, the legal sector and the executive orders from the president. It appears that communication practices are among the major obstacles to providing comprehensive adolescent sexual and reproductive health services. Participants described the presence of a lack of clarity in terms of policies:

The government policies … Should stay clear. They should not act as a barrier, for the adolescent to get the sexual reproductive health [services]. [Interview 8, medical doctor]

The participants expressed concerns about the legacy of mandatory pregnancy testing for adolescents in schools, without students’ consent or opportunity to decline; such practices persist in many schools. This testing regime is causing confusion about health professionals’ ability to provide sexual and reproductive health care. While participants believe testing for pregnancy and expelling pregnant girls from schools is damaging for the adolescents’ future, it also makes their task very difficult in a community that stigmatizes adolescent pregnancy.

This climate of stigma and uncertainty further fosters uncertainty among health professionals and their mandates; for example, one participant expressed uncertainty over whether the provision of contraceptives to adolescents, especially unmarried adolescents, was even legal:

But we don’t give them [contraceptives] to prevent pregnancies, we are not allowed. So … Even in cases of access to other things to measure to prevent pregnancies, like … Even the condoms, they [adolescents] are not allowed to use it. [Interview 9, medical doctor]

The participants expressed confusion regarding a previous statement from the president about family planning, namely that women using contraceptives are lazy. It appeared to be a challenge to reconcile this statement with the provision of quality sexual and reproductive health care for adolescents:

The policies are discouraging. Because I cannot work against it. If I do that, they will sue me. […] If it could be legal, we could provide the service and then the business will be over. But if it’s illegal, some of the health care workers … They will go to the streets and tell other people. [Interview 8, medical doctor]

Health professionals revealed that these views create a climate of uncertainty and fear of breaking the law and affect the working environment at the hospital where the adolescent sexual and reproductive health programme is implemented:

Right now, if you go to the facilities and you ask about ‘what do you think about the president’s statement on family planning?’ I am sure that only few will be able to respond. To stand up and say … They are not confused, they know *exactly* that what the president said is wrong, but they can’t really say ‘what the president says is wrong’ because then you are fighting the government itself. [Interview 15, social worker]

These unclear messages from policymakers and political leaders make confidently providing sexual and reproductive health services to adolescents challenging for health professionals. Some participants claim that this confusion has limited the community initiatives required to improve access to sexual and reproductive health services for adolescents. Because of existing uncertainties, people advocating for sexual and reproductive health and rights for adolescents were perceived to work against the government:

We are talking about the fact that the government, […] don’t want so many people talking about certain kinds of services, like family planning, publicly. So, I think that now we are going to have some challenges, because now […] we are kind of working against the government. [Interview 16, programme officer]

In addition to uncertain government messages, health professionals are concerned about being responsible for bringing the community foreign ideas that challenge traditional norms. A contradiction emerges between the social norms of sexual abstinence before marriage in Tanzania and the notion of granting access to sexual and reproductive health services for adolescents:

I mean the culture and traditions are also affecting the providers because they also live in the community. […] When you want to bring something new in a culture [it] is really … A very long-time culture, and you fight … We are fighting with society. [Interview 15, social worker]

In other words, health professionals are part of the communities they operate in. Their professional practice is affected by the communities they live in, and they express difficulties in challenging these norms.

## Discussion

The results of this study show that health professionals experience various challenges in delivering effective sexual and reproductive health services for adolescents. Stigma towards adolescents seeking sexual and reproductive health care, the influence of social norms, and the struggle to provide integrated sexual and reproductive health services and challenges to include adolescent males echo findings from the literature [27,29–31,38–42]. Our analysis suggests that these findings are closely related to two issues at play in this case study: the legacy of vertical HIV programmes, which hampers the provision of integrated sexual and reproductive health services for adolescents, and the challenge for health professionals in negotiating the various messages they receive about their ability to provide sexual and reproductive health care to adolescents.

The legacy of vertical HIV programmes and continued funding of disease- or topic-specific interventions in Tanzania [43] has created a context in which the provision of integrated sexual and reproductive health and HIV care for adolescents has been difficult. This study shows that health professionals perceive testing and treatment for HIV as more accessible to adolescents compared to other sexual and reproductive health care services. Another study from Tanzania similarly reports that young people living with HIV have more access to sexual and reproductive health services and education than do other groups of adolescents [44]. Moreover, this study adds to findings suggesting that sexual and reproductive health services target pregnant women and the prevention and treatment of STIs [30,45].

The findings from this study further suggest a possible link between poor integration of service and the inability to reach adolescent males. Family planning programmes have previously been found to mostly target women, often with the perspective that women are the main contraceptive users [38,39,46]. Reasons for men’s limited use of sexual and reproductive health services have previously been related to understanding social support for men as compared to women [47], and stereotypes about masculinity [40]. In this study, health professionals echo some of these concerns. Yet, the effects of integrated sexual and reproductive health and HIV services on the health-seeking of men and adolescent males are poorly understood, and there has been a call for more research on this subject [42]. A more in-depth understanding of the relationship between the types of services provided and the needs of adolescent males would be valuable to help improve access to sexual and reproductive health services for all adolescents.

Yet, solving these challenges and being able to provide comprehensive sexual and reproductive healthcare services for both adolescent females and males is challenging in the current context. Health professionals negotiate contradictory messages with regards to adolescent sexual and reproductive health policies, the legal framework to provide health care for under-aged youth, the president’s personal views on family planning, and views from their communities. This negotiation, in turn, creates a climate of uncertainty amongst health care professionals regarding their ability to serve the adolescent population. It is likely that health professionals’ stigma and quality of care is affected by this climate of uncertainty, especially since some health professionals believe that they are breaking the law by providing adolescents with contraceptives. This uncertainty has further been amplified by the current president’s public opinion on family planning, namely that it is unnecessary and that women using contraceptives are lazy [48]. This statement may have had implications for health professionals providing family planning, especially to a more controversial group: the adolescents.

Stigma and poor attitudes towards adolescents are well documented in the literature, with examples from Tanzania [29]. Health professionals have previously been blamed for their judgmental and stigmatizing attitudes in the provision of sexual and reproductive health care [24,27,29–31]. This study suggests that the reasons for poor provision of care are not solely linked to culture and norms but are much more complex and are closely related to the socio-economic and political climate in which these health professionals practice.

Current national policy guidelines in Tanzania state that adolescents should obtain relevant and appropriate preventive, rehabilitative and curative family planning and STI services [49]. However, those standards lack specific explanations of what is deemed ‘relevant’ and ‘appropriate’ and are missing guidelines and clarity in the context of individual clients, such as unmarried adolescents and adolescents under 18 years old, whose parents may become involved [27]. This combination of unclear policies and community stigma has previously been suggested to shape health care professionals’ attitudes and behaviours in their provision of health care to adolescents [27]. Consequently, the stigma and unfriendly attitudes by health professionals providing sexual and reproductive health care described in this study are likely a result of the challenges they experience in negotiating community norms, an unclear legal framework and policies, and statements from political leaders. Despite the challenges experienced, there is willingness and recognition by health professionals of the importance of serving the adolescent group. Efforts should, therefore, be made by advocates and policymakers to create clear policy guidelines. Endorsements of these policies by community leaders is important and could be a vehicle for communicating the policy. The law should not cause confusion for health professionals, and leaders of health programmes should help health professionals gain clarity on this point.

Health programmes such as the one implemented by the ELCT should create a space for expression and discussion of the uncertainty around providing sexual and reproductive health care to help health professionals navigate the difficulties they face, for example routines for continuous discussion at the individual, community, organizational and policy levels to support health professionals as they navigate the political space and to recognize high-quality sexual and reproductive health care provision. Moreover, a more strategic alignment of health interventions and integrated sexual and reproductive health and HIV services could help overcome the medicalisation of the service and assist in reaching adolescent males. The development of evidence-based implementation programmes could help address this challenge. Lastly, further exploration of the intersection between stigma toward adolescents’ sexual and reproductive health and health policies is warranted to form future effective health care and health programmes for adolescents.

### Limitations of the study

The results of this study are limited to the setting in which the data was collected, although certain findings may be transferable; for example, the confusion over national policy is likely to be a challenge experienced by other health professionals providing sexual and reproductive healthcare to adolescents throughout the country. Only SB collected the data, so personal bias may have influenced the data collection although SB took extensive notes during the whole research process. The notes were discussed with all researchers and reflected upon to limit bias from the data collection in the results. Some of the interviews were short, which was the case in only 2 interviews, however, and a total of 16 interviews made it possible to reach data saturation. Language and interpretation constraints for three interviews may have limited the quality of the data – since no professional interpreter could be used.

## Conclusion

Adolescent sexual and reproductive health is an urgent public health matter in Tanzania. Failure to address the delivery of healthcare to this population group is likely to result in poor health outcomes, including high rates of STIs, unwanted and early pregnancies, and difficulties in accessing contraceptives and safe abortion services. This study aimed to improve the understanding of health professionals’ perceptions and attitudes to deliver sexual and reproductive health services in ELCT facilities in Tanzania. The results show that the stigma of health professionals, challenges to the involvement of adolescent males, the medicalisation of sexual and reproductive health service provision, and challenges to navigating conflicting policy messages are important themes that need to be addressed to improve the effective and sensitive delivery of health interventions for adolescents. While some of these findings echo research in this field, this paper highlights health professionals’ challenges in navigating the context in which they operate, primarily driven by their fear and uncertainty in serving the adolescent patient group, in view of an ambiguous health-policy context. Such ambiguity fosters a climate of uncertainty and stigma, which inevitably affects the effective and sensitive implementation of health programmes. This paper urges policymakers, donors and implementers of adolescent sexual and reproductive health programmes in Tanzania to address this uncertainty at the political, legal and practical level to help support health professionals in serving the healthcare needs of adolescents.

## Paper context

Previous research on health professionals’ perceptions and attitudes of sexual and reproductive health services for adolescents indicate that judgemental and stigmatizing attitudes towards adolescents hamper access to sexual and reproductive health services. This study investigated health professionals’ perceptions and attitudes of the health care provision, suggesting that reasons for poor provision of sexual and reproductive health care are complex and the perception may not always be judgmental but shaped by a wider socio-political context.

## Appendix 2 Annex 2. Topic guide

**Topic guide**

Thank you for your participation in this field study which aims to investigate health professionals’ perceptions of sexual and reproductive health care for adolescents. My name is Sara Bylund and I come from Uppsala University in Sweden. The study is in connection to the project about sexual and reproductive health and rights carried out by the Evangelical-Lutheran Church in Tanzania (ELCT) with support from the Church of Sweden.

The aim of the study is *to gain knowledge about the perceptions of health care professionals that may influence the sexual and reproductive health services for adolescents*.

- Your participation is voluntary, and you can choose to cancel your involvement at any time.
- The information that is collected during the interviews will be handled with anonymity and will only be used for scientific research. The information will only be accessible to me and my supervisors.
- I would like to record this interview because I do not want to miss any important information. Do you approve?
- Since this is a study, I would like to take some notes, also to not miss any important information. Do you approve?
- During the interview, I will ask you questions and you are encouraged to tell your own stories, there are no answers that are right or wrong. I am very interested in hearing about your experiences.
- The study will be found in an online journal, this will be informed to you about after publication.

Before we start the interview, do you have any questions?

## Appendix 3 Pre-interview questions/introduction

1a) What is your name?
2a) Are you in any relationship?
3a) Are you born in Tanzania?
4a) What is your profession?
5a) How long have you held your position?
6a) Where do you live? How far away is it from the hospital/office?
7a) Could you tell me a bit about your role and responsibilities at this hospital/office?
8a) What is your background and training for your current position?
9a) For how long have you been working with sexual and reproductive health for adolescents?

**Perceptions and previous experiences of providing sexual and reproductive health care**

1b) What do you understand about sexual and reproductive health?
2b) How did you get the knowledge that you hold about sexual and reproductive health and rights?
3b) How is your experience in meeting with adolescents about sexual and reproductive health services?
  - *Probing question*: What do you think that adolescents know about the services?
4b) Why do you think these services are needed in Tanzania?
5b) How would you describe the quality of sexual and reproductive health services for adolescents at this hospital?(*Health department*: at the hospitals where you are implementing your project?)
  - *Probing question*: How do you perceive the accessibility and availability of services for adolescents?
  - *Probing question*: Are there enough resources and material for helping adolescents that come to seek sexual and reproductive health care, at this hospital? (*Health department*: at the hospitals where the project is implemented?)
6b) What services do you think are the most relevant and important for adolescents, in terms of sexual and reproductive health and rights?

*Probing question*: Are there any services that you perceive as lagging behind/are less developed compared to other sexual and reproductive health services?

7b) In your opinion, what are the healthcare providers’ responsibilities for adolescents’ sexual and reproductive health?
8b) In your opinion, what other actors are needed in order to realise sexual and reproductive health for adolescents?

**Experiences and opinions of adolescents seeking sexual and reproductive health services**

1c) How do you experience adolescents seeking sexual and reproductive healthcare at this hospital? (*Health department*: How do you perceive adolescents seeking sexual and reproductive health care at the hospitals?)

*Probing question*: What do you think makes adolescents hesitate in seeking sexual and reproductive health care?

2c) What do you think would make adolescents feel more comfortable seeking sexual and reproductive health care?
3c) Are you able to meet the adolescents in the way they expect to be met? (*Health department*: Do you think that healthcare workers are able to meet the adolescents in the way they expect to be met?)*(If not)* What do you think could be improved? *(If yes)* Can you elaborate on what you think is an appropriate way of meeting with adolescents regarding sexual and reproductive health services?
4c) How are the ‘routines’ when adolescents come here to seek sexual and reproductive health care (at the hospital)?
*Probing question*: What happens first when an adolescent comes to seek sexual and reproductive health care?
5c) What do you think are the expectations from the adolescents, as a healthcare provider for sexual and reproductive health services?
6c) What is the difference between adolescent boys and girls in seeking and obtaining sexual and reproductive health services?
7c) What do you perceive as challenging in your work of providing sexual and reproductive health services for adolescents?
  - *Probing question*: What would you like to see changed regarding the sexual and reproductive health services for adolescents in this hospital? (*Health department*: at the hospitals?)
  - *Probing question*: If you have to choose *one* thing that you personally think is challenging to improve sexual and reproductive health for adolescents, what could that be?

I have no more questions now. Is there anything you would like to add or ask before we finish this interview? Thank you for your participation.
